# Navigating the Nexus: Lenalidomide-Associated Thrombotic Thrombocytopenic Purpura

**DOI:** 10.7759/cureus.62975

**Published:** 2024-06-23

**Authors:** Ghida Akhdar, Inemesit Akpan

**Affiliations:** 1 Internal Medicine, Piedmont Athens Regional Medical Center, Athens, USA

**Keywords:** drug induced thrombotic microangiopathy, adamts 13, thrombotic thrombocytopenic purpura, lenalidomide, multiple myeloma

## Abstract

Thrombotic thrombocytopenic purpura (TTP) is a life-threatening thrombotic microangiopathy (TMA) marked by thrombocytopenia, microangiopathic hemolytic anemia, and microvascular thrombosis leading to end-organ damage. While TTP commonly results from hereditary or acquired ADAMTS13 deficiency, its association with lenalidomide is notably rare. The link between lenalidomide and TMA is unclear and requires more studies, given the high mortality risk associated with TTP. The underlying mechanism may involve immunomodulatory effects leading to ADAMTS13 inhibitory antibody formation.

Herein, we present a case of a 56-year-old male with a history of multiple myeloma status post autologous stem cell transplant, on lenalidomide maintenance therapy for over five years, who presented with progressive weakness, jaundice, palpitations, and paraesthesia in his extremities. On arrival, the patient was afebrile and was neurologically intact except for the subjective paraesthesia. He was found to have critically low ADAMTS13 activity at <0.03 IU/mL and detectable ADAMTS13 inhibitors, with other findings of TTP, requiring discontinuation of lenalidomide, in addition to receiving treatment for this life-threatening TMA.

## Introduction

Thrombotic thrombocytopenic purpura (TTP) is a life-threatening thrombotic microangiopathy (TMA) syndrome that is characterized by thrombocytopenia, microangiopathic hemolytic anemia, and microvascular thrombosis resulting in end organ damage such as acute kidney injury, myocardial infarction, and neurological symptoms [1]. TTP can result from hereditary ADAMTS13 deficiency or through an acquired deficiency of ADAMTS13 through the formation of anti-ADAMTS13 antibodies that result in platelet adhesion and aggregation [2]. Lenalidomide, an immunomodulatory drug, can be used in the treatment of certain lymphomas and multiple myeloma (MM), as is the case for our patient [3]. Although pancytopenia in the setting of myelosuppression is a well-known side effect of lenalidomide, there are very few case reports of lenalidomide-associated TTP, as seen in our 56-year-old patient with IgG lambda MM in remission on lenalidomide for maintenance therapy.

## Case presentation

A 56-year-old man with a known history of ankylosing spondylitis, history of deep venous thrombosis, and pulmonary embolism, on chronic anticoagulation with rivaroxaban, IgG lambda with large plasmacytoma in the right hip diagnosed seven years ago followed by induction chemotherapy and autologous stem cell transplant about six years ago. He achieved complete remission and was maintained on lenalidomide 10 mg daily for five years. 

**Figure 1:**
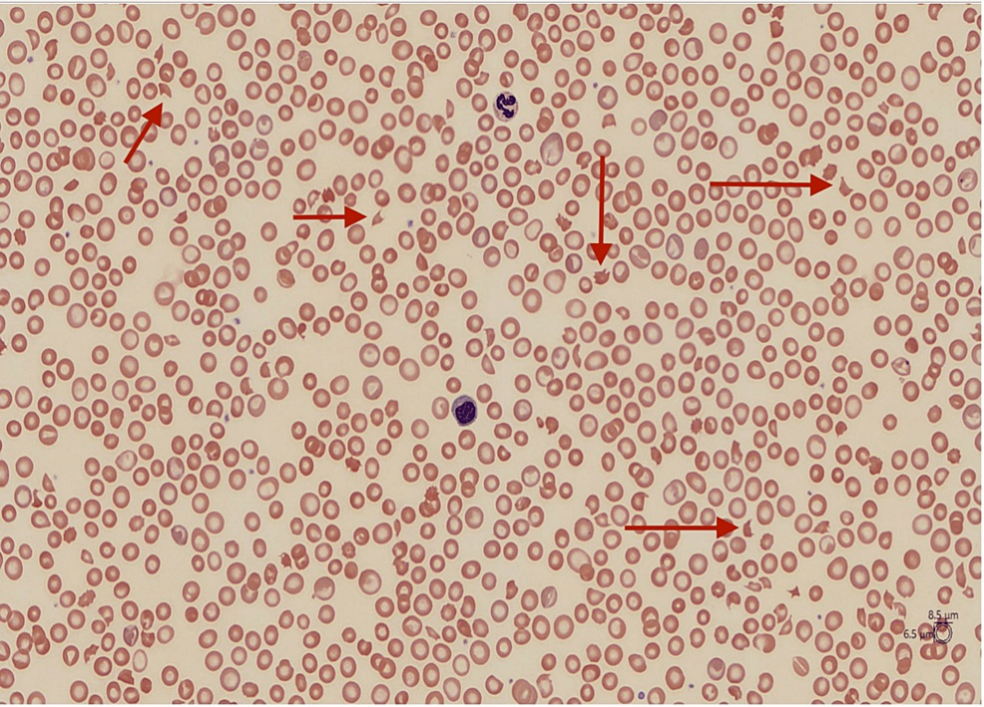
Blood smear showing moderate schistocytes

The patient presented to the hospital with symptoms of progressive weakness, shortness of breath, jaundice, palpitations, and paraesthesia involving his extremities and mouth over two to three days. He had no history of recent infections, diarrhea, or any signs of bleeding. Vitals on admission showed a temperature of 98.7°F, heart rate of 69 bpm, respiratory rate of 25 breaths per minute, blood pressure of 140/68 mmHg, and 100% saturation at room air. The physical exam was unremarkable on arrival. Laboratory findings showed hemoglobin of 7 g/dL, mean corpuscular volume (MCV) of 103.8 fL, platelets of 3 x 10^3/µL, bilirubin of 5 mg/dL, creatinine of 1.03 mg/dL, aspartate aminotransferase (AST) of 45 U/L, alanine aminotransferase (ALT) of 73 U/L, haptoglobin <8 mg/dL, elevated reticulocyte count at 15.3%, lactate dehydrogenase (LDH) of 498 U/L, and schistocytes on peripheral smear (Figure 1) were highly concerning for microangiopathic hemolytic anemia. Further laboratory investigations showed a partial thromboplastin time of 40 seconds, prothrombin time (PT) of 26.7 seconds, an international normalized ratio (INR) of 2.4, and fibrinogen of 499 mg/dL. C3 and C4 levels were normal. HIV and hepatitis testing were negative. Vitamin B12 was normal. Ferritin is slightly elevated. IgG and IgA levels were normal. Serum protein electrophoresis showed no monoclonal proteins, and immunofixation showed a few tiny oligoclonal bands in the gamma region, which are non-specific. Bone marrow flow cytometry showed a slightly increased number of B cell precursors at 4.1%, which favors hematogones. There was no flow cytometric evidence for an abnormal mature B lymphoid or myeloid population. No significant plasma cell proliferation was observed either. ADAMTS 13 activity was low at <0.03 IU/mL. An ADAMTS 13 inhibitor was detected at 0.6 (normal reference <0.4).

Of note, his most recent laboratory investigation two months prior to his admission demonstrated normal cell blood count and complete metabolic panel. 

Given the severe thrombocytopenia and anemia, along with significant schistocytes on smear, there was a concern for TMA, with consideration for immune-mediated versus drug-induced TTP. His lenalidomide was held on admission, and he received about two units of packed red blood cells during his hospitalization. Nephrology and oncology specialists were consulted, and the patient was started on daily plasma exchange and high-dose steroids, with methylprednisolone 1 g for three days, which was then switched to prednisone 1 mg/kg orally daily once his platelets started improving. He wasn’t started on platelet transfusion due to the high risk of associated thrombosis and mortality in TTP. He received two doses of rituximab 375 mg/m² within a week, with significant improvement in his platelet count and hemolysis panel. Despite the improvement in platelet count, it remained stable in the low 100s x 10^3/µL, raising concern that rituximab was not fully effective due to its removal by plasma exchange. For this reason, he was initiated on caplacizumab 11 mg intravenously once followed by 11 mg subcutaneously daily, along with plasma exchange. His hemoglobin over the course of his stay improved to 11.1 g/dL, and his platelets improved to 156 x 10^3/µL. He improved clinically and was discharged on daily caplacizumab, a prednisone taper, and plasma exchange every other day. 

## Discussion

TTP is a rare diagnosis that should be suspected promptly because, if left untreated, mortality rates are as high as 90% [4]. It is clinically suspected in the setting of microangiopathic hemolytic anemia, thrombocytopenia, and microvascular thrombosis, potentially causing neurological deterioration and renal failure, but the classic pentad presentation is very rare, accounting for 5% of cases [5].

TTP arises due to a decrease or absence in the functioning of the ADAMTS13 enzyme, and it can manifest in two forms: congenital or acquired. The acquired form is more prevalent, typically triggered by the body's production of autoantibodies that target the ADAMTS13 enzyme. Multiple drugs have been associated with immune-mediated TMA, most notably chemotherapy agents such as gemcitabine, mitomycin, and certain antibiotics. In contrast, the congenital form of TTP is less common and arises from genetic mutations affecting the ADAMTS13 enzyme. Despite these insights, the precise risk factors contributing to the formation of inhibitory autoantibodies against ADAMTS13 remain to be clearly defined [5,6]. Our patient had critically low ADAMTS13 activity at <0.03 IU/mL and detectable ADAMTS13 inhibitors, which confirmed the diagnosis.

Targeted therapy with lenalidomide is the mainstay of MM maintenance treatment after autologous stem cell transplant. In the management of MM, proteasome inhibitors are known to be a cause of TMA, even though only a few cases were found in the literature [7-10].

The mechanism of action of lenalidomide in MM is not well understood but may relate to tumor necrosis factor (TNF)-alpha inhibition, downregulation of proinflammatory cytokines, direct cytotoxic effects on myeloma cells, and antiangiogenic activity. Another theory that can be explored includes in vitro findings that lenalidomide inhibits the vascular endothelial growth factor (VEGF) pathway in a dose-dependent manner, leading to the inhibition of endothelial cell formation and angiogenesis [11]. In the kidneys, low VEGF can trigger microvascular injury and induce TMA [12]. Another possible theory is through the transient increase of factors VIII and von Willebrand factor, in addition to protein C resistance, which increases the risk of thrombosis, but this is expected to be transient and occurs only with initiation of immunomodulators, which was not the case for our patient and most of the previously reported cases [13]. It could also be that lenalidomide induces the development of anti-ADAMTS13 antibodies. Recently, three cases were reported where anti-ADAMTS13 antibodies were identified in addition to a positive response to rituximab and total plasma exchange (TPE), suggesting an underlying immune-mediated reaction [14]. Autoimmune responses, such as autoimmune cytopenia with lenalidomide use, are mostly reported to occur in the first few months of treatment [15].

Just like in other reported cases, we can appreciate a similarity in our patient’s presentation, including creatinine less than 2 mg/dL, platelet counts less than 30 × 10^3/µL, and an intermediate risk PLASMIC score. Interestingly, upon literature review, in the first case reported by Cheah et al., the patient had a medical history of rheumatoid arthritis. Upon contacting the author of the most recent case series, two out of the three reported patients had a history of autoimmune disorders [16]. So, it is unclear if a history of autoimmune disorders is a risk factor for lenalidomide-triggering immune-mediated TTP. 

Of the few reported cases of lenalidomide-associated immune thrombocytopenia, interestingly, there are a handful of reports of thrombocytopenia persisting months after drug discontinuation, despite the rapid renal clearance of lenalidomide [6,17,18]. Our patient continues to require maintenance plasma exchange, rituximab, caplacizumab, and prednisone taper to maintain his platelet count over the past two weeks since he was discharged from the hospital. This therapeutic regimen led to significant clinical and hematological improvement, with the patient's hemoglobin and platelet levels returning close to normal ranges. Further investigations are required on the mechanism behind lenalidomide-induced TTP and ways to better manage it to help improve patient outcomes.

## Conclusions

This case underscores the necessity for vigilance regarding TTP as a potential adverse effect of lenalidomide, despite its rarity. The link between lenalidomide and TMA is unclear and requires more studies, given the high mortality risk associated with TTP. The underlying mechanism may involve immunomodulatory effects leading to ADAMTS13 inhibitory antibody formation. Evidence is growing in support of an underlying pathophysiology behind lenalidomide and TTP. Providers should be aware of this rare but serious risk and be more vigilant in patients with rheumatological disorders. Prompt recognition and comprehensive treatment are crucial to improving patient outcomes in such cases.
